# Characteristics of worry in Generalized Anxiety Disorder

**DOI:** 10.1016/j.jbtep.2013.03.004

**Published:** 2013-12

**Authors:** Colette R. Hirsch, Andrew Mathews, Belinda Lequertier, Gemma Perman, Sarra Hayes

**Affiliations:** a King's College London, UK; b University of Western Australia, Australia; c University of California, Davis, USA

**Keywords:** Generalized Anxiety Disorder, Panic Disorder, Worry, Attentional control, Metacognitive beliefs

## Abstract

**Background & objectives:**

Groups of clients and community volunteers with Generalized Anxiety Disorder (GAD) and clients with Panic Disorder were compared to a group with elevated worry but without GAD on a range of measures, to identify individual differences beyond a high propensity to worry.

**Method:**

Participants completed standardised questionnaires and a behavioural worry task that assesses frequency and severity of negative thought intrusions.

**Results:**

Relative to high worriers, clients with GAD had higher scores on trait anxiety, depression, more negative beliefs about worry, a greater range of worry topics, and more frequent and severe negative thought intrusions. Relative to community volunteers with GAD, clients in treatment reported poorer attentional control. Compared to clients with Panic Disorder, clients with GAD had higher trait anxiety, propensity to worry, negative beliefs and a wider range of worry content.

**Conclusions:**

Results confirmed expectations of group differences based on GAD diagnostic criteria, but also revealed other differences in mood, characteristics of worry, and perceived attentional control that may play a role in the decision to seek treatment.

## Highlights

• People with GAD were compared with high worriers and a Panic Disorder group.
• Severity & frequency of negative intrusions was greater in GADs than in high worriers.
• GAD clients had poorer perceived attentional control than Community GADs.
• GAD clients reported a greater need to control their thinking than Community GADs.
• GAD clients had a greater range of worry content and concerns than panic clients.

## 1 Introduction

Worry is characterised by the repeated experience of thoughts about potential negative events, and reported proneness to worry varies continuously across the normal population (Ruscio, Borkovec, & Ruscio, 2001). Chronic, excessive and uncontrollable worry about multiple topics is the main defining feature of Generalized Anxiety Disorder (GAD; Diagnostic and Statistical Manual of Mental Disorders 4th Edition: DSM IV; American Psychological Association, 1994), often causing severe incapacity. In addition to excessive and uncontrollable worry, a diagnosis of GAD requires endorsement of at least three other associated symptoms (e.g., concentration problems, sleep difficulties, fatigue). However, given that excessive, uncontrollable worry is the central requirement for a diagnosis of GAD, it was the focus of the current study.

Ruscio et al. (2001) reported that worry propensity lies on a normal continuum. Individuals with GAD are characterised by the presence of severe and uncontrollable worry. Some excessive worriers without GAD also report other associated symptoms although (necessarily) not in sufficient number to meet diagnostic criteria (Ruscio, 2002). Whether or not an individual experiencing high levels of worry also meets diagnostic criteria for GAD thus depends on multiple criteria that include the presence of somatic as well as cognitive symptoms. When multiple criteria must all be met to achieve a categorical distinction, it is not clear which among them are essential, or even useful, in distinguishing between diagnosed and non-diagnosed groups. The main aim of the present study was to test hypotheses derived from the worry-related criteria currently used to diagnose GAD, by assessing the extent to which they actually distinguish individuals with this diagnosis from a non-clinical group with similarly high levels of worry, or another anxiety disorder in which worry is not thought to be central, such as Panic Disorder. Failures to find predicted differences would have potentially important implications for the clinical or theoretical usefulness of the assumed central criteria. Furthermore, other differences emerging could inform attempts to formulate a comprehensive model of GAD and the development of more effective treatments. Summarized below are the main issues and questions to be addressed in the present study.

(1) Range of worry topics. Although frequent worry about multiple topics is the central requirement for diagnosing GAD, it does not necessarily follow that the number of topics worried about actually distinguishes high worriers meeting diagnostic criteria for GAD from high worriers who do not meet all the required criteria; nor that frequency of worry distinguishes those with GAD from those with other anxiety disorders not defined in terms of the worry about many topics. We therefore explicitly tested the previously unexamined hypothesis that the range of worry topics would be greater in a group meeting diagnostic criteria for GAD than a matched high worry group not meeting these criteria or clients with Panic Disorder.
(2) Perceived and actual control. Similarly, the fact that reported lack of perceived control over worry is required for diagnosing GAD does not necessarily mean that non-GAD high worriers actually have any greater control over worry than do those with GAD. Consequently, a further hypothesis tested in the current study was that those with GAD would be less able to prevent worrisome thoughts intruding when attempting to focus their attention elsewhere, and possibly also have a more general inability to control attention, based on a self-report questionnaire designed to assess ability to control attention across a range of everyday activities.
(3) Beliefs about worry. Inappropriate beliefs about either the positive benefits or the negative consequences of excessive worry are not part of the diagnostic criteria for GAD, although some previous researchers (e.g., Ruscio & Borkovec, 2004; Wells & Carter, 2001) have found evidence suggesting that such beliefs may be both characteristic of the disorder and possibly play a part in maintaining it. Given these previous suggestions, we included a further examination of this issue using an established questionnaire measure (Meta Cognitions Questionnaire; MCQ; Wells & Carter, 2001) to test the extent to which beliefs about worry distinguish those meeting GAD diagnosis on clinical interview from equally high worriers not so diagnosed.
(4) Other emotional differences. High levels of anxiety and depression often accompany excessive worry, although again the question of whether or not such mood disturbances accompany all elevated worry states, perhaps as a consequence of worry itself, or are more likely to occur in those meeting current criteria for GAD as assessed by clinical interview has not previously been examined. It is possible that it is only the emotional symptoms that are presently required for diagnosis of GAD which distinguish those meeting diagnostic criteria for GAD from others with equally intrusive and uncontrollable worries about similarly diverse topics. We assessed this possibility by comparing GAD and matched high worriers using standard questionnaire measures of trait anxiety (State-Trait Anxiety Inventory Trait version; STAI-T; Spielberger, Gorsuch, Lushene, Vagg, & Jacobs, 1983) and depression (Beck Depression Inventory; BDI; Beck & Steer, 1987).
(5) Finally, not all those meeting criteria for GAD enter or even seek treatment and it is unclear how those not seeking help differ from similarly diagnosed groups in treatment, or for that matter from high worriers not so diagnosed. Little is known about the factors influencing individuals with similar symptoms to enter treatment or otherwise, but one obvious possibility is that those seeking treatment are experiencing greater severity in the worry-related or emotional symptoms discussed above. Another previously suggested hypothesis to be tested here is that the perceived failure of control over intrusive negative thoughts in worry is the critical factor leading high worriers to seek help (Mathews, 1990).

In earlier work, Ruscio and Borkovec (2004) addressed some (but not all) of the issues discussed above, by individually matching pairs on overall worry severity (based on their Penn State Worry Questionnaire scores; PSWQ; Meyer, Miller, Metzger, & Borkovec, 1990), with one of each pair meeting GAD criteria (as assessed using the Generalized Anxiety Disorder-Questionnaire; GAD-Q-IV; Newman et al., 2002), while the other did not. Rather than relying on reported inability to control worry, Ruscio and Borkovec (2004) used a behavioural test in which participants attended to their breathing before and after instructed worry (cf. Borkovec, Robinson, Pruzinsky, & DePree, 1983), and when signalled on four occasions participants reported if they had been distracted by a negative, positive or neutral thought at the time of the signal. Those with a GAD diagnosis (based on questionnaire) were more likely to report a negative thought than were others not so diagnosed, but only on the first occasion immediately following instructed worry. Although consistent with impaired control in GAD the effect was surprisingly short-lived, so that reported lack of control may be more perceived than real and could partly reflect stronger negative beliefs about worry in the GAD group.

Part of the present study (see Section 2 above) was similarly directed to the question of whether the characteristics of worry in clients diagnosed with GAD differ from those in a group of volunteers matched on overall reported worry severity. However, to further examine whether these groups differed in perceived or real ability to control thoughts (or both) we included a questionnaire measure of perceived control (Attentional Control Scale: Derryberry & Reed, 2002), and increased the frequency of thought samples in the behavioural worry measure to enhance sensitivity to actual control differences. Thought intrusions were also categorised in terms of valence by an assessor who was not informed about group membership to determine if negative intrusions were objectively more common in the diagnosed groups. Negative intrusions were also categorised by an assessor in terms of severity to assess whether people with GAD reported particularly negative thoughts. In addition, we distinguished between those in treatment for GAD and a community sample meeting GAD criteria (using the structured clinical interview for DSM-IV in addition to the GAD-Q-IV used by Ruscio & Borkovec, 2004 with their student sample) who were not seeking treatment.

We also contrasted those in treatment for GAD with a group being treated for Panic Disorder, to determine whether any differences found applied generally to all those seeking treatment, rather than being specific to GAD. Clients with Panic Disorder are concerned about the potential occurrence of future panic attacks, so they may worry frequently about this specific issue. They are, however, less likely to worry about a wide range of worry topics, or be as concerned about the process of worrying itself, when compared to clients with GAD. Hence, inclusion of this latter group also allowed us to test the assumption that GAD (compared to panic disorder) is associated with a greater *range* of worries, and greater *concern* about worrying, but not necessarily with a higher *frequency* of worrying. Finally, to check whether groups also differed in mood state we also obtained measures of anxiety and depression at the time of testing.

## 2 Method

### 2.1 Participants

Participants comprised 32 clients in treatment for GAD, 24 clients in treatment for Panic Disorder, 28 community volunteers who met criteria for GAD but who were not currently seeking treatment, and 35 community volunteers reporting equivalent levels of worry to the GAD groups, but who did not meet criteria for GAD. Both GAD and Panic Disorder clients were receiving a recognised treatment (e.g., medication or psychological therapy) or were on a waitlist (three clients in the GAD group and one in the Panic Disorder group). They were recruited via either the South London & Maudsley National Health Service Foundation Trust or advertisements for volunteers who were in treatment for GAD or Panic Disorder. To be included in the GAD group, on the day of testing participants had to meet current diagnostic criteria for GAD on GAD-Q-IV (in keeping with Ruscio & Borkovec, 2004) and additionally on the Structured Clinical Interview for DSM-IV Axis I Disorders interview (SCID; First, Spitzer, Gibbon, & Williams, 1996) to ensure that participants in the GAD group met criteria on clinician interview. Assessors included other sections of the SCID-I as necessary to ensure that excessive worry was evident for multiple worry topics unrelated to other Axis I disorders. Panic Disorder participants met criteria for Panic Disorder as assessed by SCID interview on the day of testing. No participants in the Panic Disorder group met criteria for GAD, but 12 being treated for GAD also had Panic Disorder.^1^ Participants in the community GAD or high worry groups were recruited via an advertisement asking for volunteers for a study of worry. The community GAD group met criteria for GAD on the GAD-Q-IV and SCID interview on the day of testing but were not currently in or awaiting treatment. Four participants in the community GAD group also met criteria for Panic Disorder. Participants were selected for the high worry group if they scored 56 or higher on the PSWQ, but did not meet criteria for GAD (or Panic Disorder). Molina and Borkovec (1994) reported that a score of 56 fell one standard deviation below the mean for individuals diagnosed with GAD.

### 2.2 Materials

#### 2.2.1 Generalized Anxiety Disorder Questionnaire (GAD-Q-IV)

The GAD-Q-IV is a self-report diagnostic measure of Generalized Anxiety Disorder. Newman et al. (2002) reported good test-retest reliability, convergent and discriminant validity, and a high level of diagnostic agreement with a clinical assessor on the ADIS (Brown, Di Nardo & Barlow, 1994).

#### 2.2.2 Penn State Worry Questionnaire (PSWQ)

The PSWQ is a 16-item questionnaire measure of trait worry (e.g., “Once I start worrying, I can't stop”), each with a 5-point answer scale from 1 (*not at all typical of me*) to 5 (*very typical of me*) yielding a total score between 16 and 80. The PSWQ has good psychometric properties in student, community, and clinical samples, with high internal consistency, short-term retest reliability, and convergent and criterion related validity (Brown, Antony, & Barlow, 1992; Davey, 1993).

#### 2.2.3 State-Trait Anxiety Inventory-Trait Version (STAI-T: Spielberger et al., 1983)

The STAI-T assesses trait anxiety and consists of items assessing 20 anxiety symptoms rated for frequency of occurrence. The STAI-T has good internal consistency and test-retest reliability (Barnes, Harp, & Jung, 2002).

#### 2.2.4 Beck Depression Inventory (BDI: Beck & Steer, 1987)

Depressive symptoms were measured using the BDI, consisting of 21 questions rated according to how participants have been feeling during the past week. Scores range between 0 and 63. The BDI has good internal consistency and test-retest reliability (Beck, Steer, Ball, Ranieri, 1996).

#### 2.2.5 Attentional Control Questionnaire (ACQ: Derryberry & Reed, 2002)

The ACQ is a 20-item questionnaire designed to assess attentional control of executive functions (e.g., “*When concentrating I ignore feelings of thirst or hunger*”; “I *can quickly switch from one task to another*”, “*I can become interested in a new topic very quickly when I need to*”). The scale has good test retest reliability (Reinholdt-Dunne, Mogg & Bradley, 2009).

#### 2.2.6 Short form of the Worry Domains Questionnaire (WDQ: Stöber & Joormann, 2001)

The WDQ is a short 10-item version of the Worry Domains Questionnaire (Tallis, Eysenck, & Mathews, 1992) that assesses worry content. It has good internal consistency (Stöber & Joormann, 2001) and there are five subscales; labelled aimless future, financial, work, lack of confidence, and relationships.

#### 2.2.7 Meta Cognitions Questionnaire-30 (MCQ: Wells & Cartwright-Hatton, 2004)

The MCQ is a 30-item version of the Meta Cognitions Questionnaire (Cartwright-Hatton & Wells, 1997), designed to assess beliefs about worry, and has good internal consistency (Spada, Mohiyeddini & Wells, 2008). There are five sub-scales: negative beliefs about worry; need for control over thoughts; cognitive confidence; positive beliefs about worry; cognitive self consciousness.

#### 2.2.8 Structured Clinical Interview for DSM-IV Axis I Disorders (SCID-I)

The SCID-I is a clinician administered semi-structured diagnostic interview used to classify DSM-IV Axis I disorders which has been shown to have high levels of inter-rater and test-retest reliability (Zanarini et al., 2000). The GAD and Panic Disorder sections of the SCID were administered in the current study. Other sections of the SCID were administered as necessary to ensure that excessive worry about multiple topics (a criterion for GAD) was not confined to concerns associated with another Axis I disorder.

### 2.3 Worry task

This task was developed by Hayes, Hirsch, Krebs and Mathews (2010), and adapted from Borkovec et al. (1983) and Ruscio and Borkovec (2004). There were three phases: a 5-min period with participants instructed to focus on their breathing, a 5-min period of worrying; and a 5-min post worry breathing focus period. During each breathing focus period, 12 tones were presented at random intervals of 20–30 s (Donaldson, 2004), signalling participants to report if their attention was focused on their breathing, or if they were experiencing a thought intrusion. If the latter, they indicated whether it was positive, benign, or negative, and provided a brief description (e.g., “*positive – going on holiday*”).

After the pre worry breathing focus period, participants identified a current worry topic related to a potentially negative future situation. They were then asked to worry about this for 5 min, and the experimenter left the room. After 5 min, the experimenter returned, and the post worry breathing focus period was completed.

Finally, the experimenter read aloud the participant's summary of each intrusion and asked them to describe what was going through their minds at the time. Descriptions were recorded^2^ for later rating by a psychologist, who assessed the valence of each intrusion (positive, neutral or negative) and, for intrusions that were categorised as negative, how negative the intrusion was on a scale of low, medium or high negativity.^3^ Another psychologist also rated the thought expansions of 20 participants' drawn randomly in equal numbers from all groups. Neither assessor was informed about group allocation, nor when the intrusion occurred. Inter-rater reliability for valence ratings using Cohen's Kappa statistic (*k*) for the valence ratings was .80 and for negativity ratings it was .73.

### 2.4 Procedure

All participants first completed a consent form and then the STAI-T, PSWQ, BDI, GAD-Q-IV, WDQ, MCQ and ACQ were given in random order. Following this the Worry Task was administered, and then the SCID was administered by a clinical psychologist. Finally participants were debriefed, thanked for their time, and paid £20 ($30).

## 3 Results

### 3.1 General characteristics

There were 17 males and 15 females in the clinical GAD group, 9 males and 15 females in the Panic Disorder group, 14 males and 14 females in the community GAD group, and 12 males and 23 females in the high worry group. The groups did not differ significantly in terms of gender distribution, *χ*^2^ (3, *N* = 119) = 3.24, *p* = .36. Average age was 39.72 years (SD = 12.19) with no significant difference between groups, *F* (3,115) = 1.15, *p* < .333, partial *η*^2^ = .03. Average years of education was 14.38 (SD = 2.15) with no significant difference between groups, *F* (3, 115) = .38, *p* = .77, *Partial η*^2^ = .01.

A one-way analysis of variance with a between-subjects factor of group (high worry vs. GAD community vs. GAD clinical vs. Panic Disorder) was performed on PSWQ scores. This analysis revealed a significant effect of group, with high worry, community GAD and clinical GAD groups all demonstrating higher scores than the Panic Disorder group. As planned, since the high worry group was selected to not differ from GAD norms, the other three groups' PSWQ scores did not significantly differ. See Table 1 for means, standard deviations and statistics.

### 3.2 Worry task

A repeated measures ANOVA was performed on the number of negative intrusions during the two breathing-focus periods, with one between-subjects factor, group (high worry vs. GAD community vs. GAD clinical vs. Panic Disorder), and two within-subject factors, time (pre- vs. post-instructed worry), and rater (self vs. assessor). See Table 2 for means and standard deviations. There were no main effects of rater, *F* (1,100) = 1.12, *p* = .293, *Partial η*^2^ = .01, or time, *F* (1,100) = 1.35, *p* = .274, *Partial η*^2^ = .013, nor any interactions between time and group, *F* (3,100) = 1.51, *p* = .217, *Partial η*^2^ = .043, rater and group *F* (3,100) = 1.13, *p* = .341, *Partial η*^2^ = .033, or time, rater and group, *F* (3,100) = .62, *p* = .602, *Partial η*^2^ = .018. There was only one significant finding; a main effect of group, *F* (3,100) = 3.05, *p* = .032, *Partial η*^2^ = .084. Post hoc pairwise comparisons, corrected for multiple comparisons, indicated that high worriers had significantly fewer negative thought intrusions than both community GAD participants, *M* = 1.86 vs. *M* = 3.27, *p* = .045, and clinical GAD participants, *M* = 1.86 vs. *M* = 3.31, *p* = .045. No other post hoc tests reached significance (clinical GAD vs. Panic *p* = .224; community GAD vs. panic *p* = .224; clinical GAD vs. community GAD *p* = .949; panic disorder vs. high worry *p* = .409).

Chi-square analysis of assessor categorisations of participant's negative intrusions as being of low, medium or high negativity indicated that these were less negative in high worriers than those in the diagnosed groups (community GAD, clinical GAD or Panic Disorder), *χ*^2^ (6, *N* = 98) = 26.92, *p* < .001. Thus, despite being matched for PSWQ scores, actual samples of thought content suggest that negative intrusions were more frequent and more negative in those with a GAD diagnosis than high worriers.

### 3.3 Worry-related questionnaire measures

To investigate the effect of group membership on the WDQ, MCQ and ACQ data, a multivariate analysis of variance with a between-subjects factor of group (high worry vs. GAD community vs. GAD clinical vs. Panic Disorder) was performed on the questionnaire data. The main effect of group was significant, *F* (9, 270) = 3.00, *p* = .002; *Partial η*^2^ = .07. Looking at the questionnaires separately, all effects were significant. See Table 3 for means, standard deviations and statistics.

#### 3.3.1 ACQ

Multiple comparison corrected post hoc tests for the ACQ revealed that clinical GAD clients had poorer perceived control, as indicated by lower scores on the ACQ, than community GAD participants, with no other significant group differences on this measure.

#### 3.3.2 WDQ

In similar analyses of WDQ scores, the clinical GAD group scored more highly than both the panic disorder and high worry groups, but the other group comparisons on the WDQ were not significant.

While high scores on the WDQ indicate that the person endorses worries as being relatively *frequent* overall, they do not necessarily reveal the *range* of different worry domains highly endorsed. This is particularly relevant here because a diagnosis of GAD requires worry to be about multiple topics, although it remains uncertain if this implies a greater actual range of topics than in other groups. Domains differ in the frequency that items are endorsed, so each domain requires its own threshold to be able to determine whether a particular participant endorsed the given domain highly for this population. Consequently, the range of worries was instantiated by computing an index reflecting the number of different domains about which each individual reported worrying relatively frequently compared to the study population. First, we computed the median frequency of worry for each domain across the entire population. Scores at or above the median level of worry were then taken to indicate a relatively high frequency of worry about that domain. The total number of domains reaching median levels or above for each individual was then used as the index of the extent to which worries were spread across many domains. A univariate ANOVA performed on the index data showed a significant effect of group, *F* (3,115) = 4.78, *p* = .004, *Partial η*^2^ = .11. Post hoc tests, corrected for multiple comparisons, indicated that the clinical GAD group scored more highly than both the panic disorder and high worry groups (*p* < .006 and *p* < .006 respectively), but the other group comparisons were not significant (community GAD vs. high worry *p* = .232; panic disorder vs. high worry *p* = .677; clinical GAD vs. community GAD *p* = .171; community GAD vs. panic disorder *p* = .171).

#### 3.3.3 MCQ

The clinical GAD group scored more highly than both panic disorder and high worry groups. The other group comparisons on the MCQ were not significant.

Further analysis was conducted comparing the groups on the subscales of the MCQ (positive beliefs, negative beliefs, cognitive confidence, need for control of thoughts and cognitive self-consciousness). Examining each subscale individually revealed significant effects for negative beliefs, need to control thoughts and cognitive confidence, see Table 4 for means, standard deviations and statistics.

For negative beliefs about worry, the clinical GAD group scored more highly than both panic disorder and high worry groups, but the other group comparisons were not significant. The need for control of thoughts subscale showed significant differences between clinical GAD and all other groups, with no other group difference reaching significance. The clinical GAD group also demonstrated significantly greater difficulties with cognitive confidence than the Panic Disorder group, with all other comparisons failing to meet significance.

### 3.4 Emotion questionnaires

A multivariate analysis of variance with a between-subjects factor of group (high worry vs. GAD community vs. GAD clinical vs. Panic Disorder) was performed on the scores from the emotion questionnaires (STAI-T & BDI; see Table 1 for means, standard deviations and statistics). The main effect of group was significant, *F* (6, 224) = 9.37, *p* < .000; *Partial η*^2^ = .20. Looking at the questionnaires separately, the main effect of group was significant for both STAI-T, and the BDI. Pairwise comparisons, corrected for multiple comparisons, showed that for STAI-T the clinical GAD group had significantly higher STAI-T scores than the all other groups. Furthermore, the community GAD group had higher STAI-T scores than the high worriers and the Panic Disorder group. The high worriers and Panic Disorder groups did not significantly differ on STAI-T scores. In relation to BDI scores the GAD clinical groups scored significantly higher than high worriers, but other group comparisons did not reach significance.

## 4 Discussion

### 4.1 Differences between high worriers with and without GAD

Consistent with expectation and previous observations, we found that clients with GAD reported more negative beliefs about worry, lack of cognitive confidence, and need to control thinking, than did high worriers not meeting diagnostic criteria (Wells & Carter, 2001). Other expected - but not previously tested - findings from present study included the greater range of worry topics on the Worry Domains Questionnaire in those with GAD, and higher levels of anxious and depressed mood than in high worriers not meeting GAD criteria. In a further previously unexplored contrast of self-report questionnaires, we found that community volunteers with GAD (but not in treatment) differed from non-diagnosed high worriers only in reporting higher trait anxiety.

In addition to the presence of anxiety symptoms and the generality of worry, diagnosis of GAD requires that worry is *perceived* to be uncontrollable. We investigated whether worry was *actually* more uncontrollable in those with GAD than high worriers by assessing negative intrusions before and after a period of instructed worry. High worriers had fewer negative thought intrusions than individuals with GAD (both clinical and community groups), as judged by participants themselves or, as assessed for the first time here, by an objective assessor. This extends Ruscio and Borkovec's (2004) finding that high worriers differ from those with GAD in the frequency of negative thought intrusions, given that the earlier finding was limited to a single self-rated sample immediately after instructed worry in university students. Furthermore, for the first time it was established that the negative intrusions reported by people with GAD (clinical and community groups) are independently judged as more negative in content than those reported by high worriers.

Our findings support current diagnostic criteria insofar as those with a GAD diagnosis were more anxious, less able to control worry (as assessed by the number of negative thought intrusions) in comparison with a non-diagnosed group matched for reported propensity to worry. However, when comparing only the GAD sub-group currently in or awaiting treatment with high worriers, differences emerged that are not captured by the diagnostic criteria alone: the clinical GAD group reported more depression, greater need to control thinking, more negative beliefs about worry, less cognitive confidence, a greater range of worries and the content of their negative intrusive thoughts were judged to be more emotionally negative.

### 4.2 Differences between GAD groups associated with seeking treatment

Another question that we sought to address for the first time was how individuals who met diagnostic criteria for GAD but who were not seeking treatment differed from those who were in treatment (or on a waitlist). As predicted, compared to the community GAD group, those in treatment reported poorer perceived attentional control and also expressed a greater need to control their thinking. Despite also differing in trait anxiety, the two GAD groups did not significantly differ in reported propensity to worry, depressed mood, extent of overall metacognitive beliefs about worry, the number or range of topics about which they worried, or in the frequency or negativity of thought intrusions. In sum, it appears that there are some factors beyond those specified in the diagnostic criteria that distinguish those with GAD diagnoses who are currently receiving treatment from those who are not. Because treatment is available without charge within the UK via the National Health Service, we suppose that the differences we found may reflect factors influencing the treatment-seeking process (rather than economic considerations). Differences observed in the present study include the finding that GAD clients in treatment report being less able to control attention (e.g., to ignore unwanted thoughts), whilst at the same time believing that they need to control their thinking more than those not in treatment. Both of these factors could enhance the sense of being unable to cope with excessive worry (cf. Mathews, 1990, 2004). Thus, although poor *actual* control over intrusions, as assessed in the worry task, was associated with diagnostic status irrespective of treatment (i.e., high worriers had fewer intrusions than both GAD groups), the *perception* of lack of control, together with the heightened perceived importance of being able to control cognitions, may be more influential in leading individuals with a diagnosis of GAD to seek treatment. Given this, then addressing GAD clients' perceived inability to control worry early in treatment (e.g., using worry free zones to enable clients to develop a sense of control over worry; Borkovec & Sharpless, 2006), whilst being aware of, and sensitive to, any beliefs that it is important to control their thoughts, may be particularly helpful. Future research could focus on determining what factors differentiate treatment seeking and non-treatment seeking individuals with GAD, once clinical severity is taken into account.

### 4.3 Differences between clients with Panic Disorder and GAD

In a novel comparison between GAD and those with Panic Disorder, we found that GAD clients reported higher trait anxiety, greater propensity to worry, more pervasive worry and more metacognitive beliefs about worry. As expected, given that the diagnostic criteria for GAD include worry about multiple topics, the GAD group reported excessive worry across a greater number of domains than did the Panic Disorder group. GAD clients also reported more negative beliefs about worry, greater need to control thoughts and less cognitive confidence than Panic Disorder clients. However, the groups did not significantly differ in terms of depressed mood, perceived attentional control, the number of negative intrusions, or in terms of how negative the negative intrusions were. The major differences found thus reflected greater concerns about worry (and a greater range of worry topics) in GAD, rather than any apparent difference in the actual frequency or negativity of thought intrusions.

### 4.4 Limitations

There are a number of limitations of the study. The worry task was always administered after the questionnaires and it is possible that this might have led to more negative thought intrusions due to priming by questionnaires in some participants. In future research it would be preferable to randomise the task and questionnaire order. Furthermore, the use of self-report scales to assess group differences may be problematic in its own right. As has been documented elsewhere (Nisbet & Wilson, 1977) self-report can be based on post hoc rationalisations and so may be less valid than behavioural measures. Indeed, in the current study high worriers were better able to focus attention on their breathing than those with GAD, but their self-reported attentional control assessed by questionnaire did not differ. A similar discrepancy between ACQ questionnaire and a behavioural measure of visual attention in trait anxious individuals has been reported by Reinholdt-Dunne et al. (2009), indicating this issue warrants further investigation. However, as noted above, the *perception* of poor control – irrespective of its validity – may in itself have important deleterious consequences, such as raising emotional concerns and the desire to seek help.

High worriers reported less depressed mood than the clinical GAD group, so it is possible that depression (rather than GAD *per se)* may explain some of the differences between these groups. Further research that assesses caseness for depression will be needed to investigate this possibility. However, despite the differences in depressed mood between high worriers and clients with GAD, the community GAD group and high worriers did not differ significantly in terms of depression. Consequently GAD diagnosis, more than depression, appears to be associated with the greater frequency of negative intrusions, and their greater negativity, seen in GAD than in high worriers.

As is typical of GAD, where co-morbidity is very common, the clinical GAD group included a number of individuals who also had co-morbid panic disorder. Analyses run comparing clients with GAD who had comorbid panic disorder with those who did not showed no significant differences. However, the fact that a number of clients with GAD had comorbid panic disorder needs to be borne in mind when considering the comparisons between clinical GAD and Panic Disorder groups.

## 5 Conclusions

In summary, the current study found evidence to support the specificity and utility of the GAD diagnostic criteria for differentiating clients with GAD from high worriers and clients diagnosed with Panic Disorder, as well other factors that differentiate treatment-seeking GAD clients from a community sample of individuals meeting GAD criteria. A number of differences were found to contribute to the differential diagnosis of GAD or Panic Disorder, including some that were expected given that diagnostic criteria for GAD focus on excessive worry about a wide range of topics. However, the number of negative intrusions in the worry task, as assessed independently, did not significantly differ between these clinical groups. Although future research is required to investigate generalization of the worry task to everyday situations, such findings suggested that, compared to Panic Disorder, clients with GAD have greater concern about their inability to control worry and its perceived negative effects (e.g., worry about worry; Wells, 1995), rather than there being differences in actual frequency or negativity of worry-related intrusions.

Of particular interest were the distinctions found between high worriers and those with a diagnosis of GAD that support the assumption that those with GAD do experience a real excess of uncontrolled intrusive thoughts having more negative content, beyond that experienced by a high worry group reporting the same elevated propensity to worry. These findings suggest that the transition from high worry to diagnosable GAD is associated with an objective (assessor-rated) elevation in the distressing and intrusive properties of intrusive thoughts. In sum, the main factors assessed here that differentiated those with GAD (clinical or community) from high worriers without GAD were elevated trait anxiety and reduced ability to prevent particularly distressing thoughts from intruding into awareness (in the behavioural worry test). Given this, clinicians could consider including the behavioural worry task in clinical assessment of GAD. This would provide important information about actual lack of controllability of worry, over and above perceived uncontrollability provided by self-report. Furthermore, the worry task could also be included at the end of treatment to determine whether clients have indeed gained better behavioural control over worry, as would be needed to no longer meet criteria for GAD.

The other noteworthy set of findings concerned the division of those with GAD according to their clinical status, that is, whether they were patients in or awaiting treatment, or were members of the community who were not seeking treatment. When only the clinical GAD group was compared with high worry participants, some additional differences in beliefs about worry emerged, although these were confined to its negative attributes, perhaps reflecting the perception of inability to control negative thoughts. Importantly, while this group of clients in treatment for GAD differed from high worriers without GAD in a number of other respects (such as low mood, and range of worries), individuals with GAD who were *not* currently seeking treatment did not significantly differ from high worriers in the same ways. This contrast suggests that differences found between clinical and non-clinical control groups (in this case clinical GAD vs. high worriers) that could be attributed to diagnostic status alone, may in fact be associated with treatment seeking status rather than diagnosis *per se*.

Similarly, although diagnosis of GAD (clinical or community groups vs. high worriers) was not associated with differences in perceived inability to control attention (as assessed by ACQ), this factor did appear to contribute to the distinction between GAD clients currently in treatment and individuals with GAD who are not currently seeking treatment. Interestingly, therefore, rather than a further increase in the factors associated with a GAD diagnosis itself (such as a greater number of highly negative intrusions), it may be the *perception* of inability to control worry, together with the belief that it is important to control such thinking, that leads those with GAD to seek treatment. This implies that there may be important characteristics of those who find worry intolerable and thus seek treatment that are not captured by the diagnostic criteria alone. The differences between community and clinical GAD populations (and their respective differences from high worriers) could be taken into account when considering the best way to intervene clinically with individuals with GAD who seek treatment. For example, the factors leading to seeking help may be directly addressed by techniques that enable the client to realise they do have some control over worry, such as the use of negative thoughts as a cue to postpone worry and substitute more positive thoughts or activities. Treatment decisions of this sort can be guided by assessing both beliefs about control and actual control of intrusions in a behavioural worry test.

Finally, we suggest that a complete account of causal factors in pathological worry needs to take the individual differences identified here into account, including the possible roles of perceived control. In a recent cognitive model of pathological worry (Hirsch & Mathews, 2012) we reviewed evidence supporting the interactive roles of habitual processing biases favouring threat content (e.g., Hayes, Hirsch & Mathews, 2010; Hirsch, Hayes & Mathews, 2009; Hirsch et al., 2011; Krebs, Hirsch & Mathews, 2010), and top-down attentional control (e.g., Bishop, 2009; Hayes, Hirsch & Mathews, 2008; Leigh & Hirsch, 2011) which can be used to oppose negative thought intrusions, or be captured by threatening content and lead to protracted worry. Such an interactive process is consistent with many of the present findings, but as discussed earlier, some findings suggest that a further distinction should be made between *perceived* and *actual* control. While actual control over negative thoughts distinguishes between those meeting GAD diagnostic criteria and those who do not, the perception of control seems more critical in obtaining treatment.

## Figures and Tables

**Table 1:** Mean scores and statistics for emotion questionnaires (standard deviation in parentheses).

	High worry (Group 1) *N* = 35	GAD community (Group 2) *N* = 28	GAD clients (Group 3) *N* = 32	Panic clients (Group 4) *N* = 24	*F* (df)	*Partial η*^2^	Post hoc *p < .05*
Penn State Worry Questionnaire	64.63 (6.10)	63.17 (9.35)	65.48 (7.77)	56.82 (11.55)	5.40** (3, 115)	.12	1, 2, 3 > 4
State-Trait Anxiety Inventory – Trait Version	51.24 (7.53)	56.54 (8.62)	62.75 (5.95)	50.69 (8.32)	16.67** (3, 113)	.31	3 > 2 > 1, 4
Beck Depression Inventory	13.42 (7.26)	18.37 (8.69)	21.60 (9.14)	18.25 (10.40)	4.88** (3, 113)	.12	3 > 1

Note: ** = *p* < .01.

**Table 2:** Mean number of negative thought intrusions before and after instructed worry as assessed by participants (Self) and assessor (standard deviations in parentheses).

		High worry *N* = 27	GAD community *N* = 26	GAD clients *N* = 27	Panic clients *N* = 24
Pre Worry	Self	2.04 (1.68)	2.85 (2.48)	3.07 (2.50)	2.46 (2.55)
	Assessor	1.74 (1.75)	3.31 (2.95)	2.81 (2.34)	2.58 (2.75)
Post Worry	Self	2.00 (1.57)	3.42 (2.14)	3.85 (3.26)	2.54 (2.21)
	Assessor	1.67 (1.57)	3.50 (2.66)	3.48 (3.42)	2.08 (1.64)
Average		1.86 (1.31)	3.27 (2.25)	3.31 (2.61)	2.42 (1.86)

Note: Average = average number of intrusions across pre and post worry periods, for self and assessor ratings.

**Table 3:** Mean scores and statistics for worry-related questionnaire measures (standard deviation in parentheses).

	High worry (Group 1) *N* = 35	GAD community (Group 2) *N* = 28	GAD clients (Group 3) *N* = 32	Panic clients (Group 4) *N* = 24	*F* (3, 113)	*Partial η*^2^	Post hoc *p < .05*
Attentional Control Questionnaire	47.73 (8.12)	48.60 (6.65)	42.98 (7.62)	46.02 (7.92)	3.10*	.08	2 > 3
Meta Cognitions Questionnaire	69.32 (11.49)	72.22 (12.18)	78.73 (13.66)	66.55 (17.26)	4.42**	.11	3 > 1, 4
Worry Domains Questionnaire	21.43 (9.27)	24.29 (6.70)	27.31 (6.07)	19.63 (8.87)	5.11**	.12	3 > 1, 4

Note: High scores on the Attentional Control Questionnaire indicate better attentional control. * = *p* < .05, ** = *p* < .01.

**Table 4:** Mean scores and statistics for Meta Cognitions Questionnaire subscales (standard deviations in parentheses).

	High worry (Group 1) *N* = 35	GAD community (Group 2) *N* = 28	GAD clients (Group3) *N* = 32	Panic clients (Group 4) *N* = 24	*F* (15, 304)	*Partial η*^2^	Post hoc *p < .05*
Negative beliefs	16.18 (.63)	17.89 (.70)	19.22 (.65)	16.34 (.75)	4.64**	.11	3 > 1, 4
Need for control	14.65 (.76)	12.18 (.84)	15.35 (.79)	12.13 (.91)	3.64*	.09	3 > 1, 2, 4
Cognitive confidence	12.14 (.80)	12.75 (.88)	14.34 (.82)	10.71 (.95)	2.93*	.07	3 > 4
Positive worry beliefs	11.56 (.76)	11.36 (.84)	11.53 (.78)	11.54 (.90)	.30	.01	
Cognitive self-consciousness	16.80 (.70)	18.04 (.77)	18.28 (.72)	16.83 (.84)	1.10	.03	

Note: Higher scores on the cognitive confidence subscale indicate poorer cognitive confidence; * = *p* < .05, ** = *p* < .01.

## Footnotes

1 All analyses reported below were re-run comparing Clients with GAD without Panic disorder with clients with GAD who also had Panic disorder. The effect of comorbid panic disorder was non-significant for all analyses and this did not interact with other factors, indicating that the clients with GAD and comorbid Panic did not respond differently to clients with GAD who did not have comorbid panic disorder.

2 Unfortunately, due to technical failure 15 participants' expanded descriptions were not recorded successfully and could not be rated by the assessor. While more data were lost from some groups than others, high worry 8/35 (22.9%); GAD Community 2/28 (7.1%); GAD Clinical 5/32 (15.6%); Panic Disorder 0/24 (0%), the number of participants who had recordings available for the assessor to rate were similar across groups: high worry 27; GAD Community 26; GAD Clinical 27; Panic Disorder 24.

3 Assessors were instructed to rate how negative the content of the thought intrusion was. Assessors were provided with training and practice on how to use the scale along with examples of low (tired; bored; itch; cold; hot; late), medium (finances; work/study issues; minor relationship break-up), and high (serious illness; attack; death; major relationship break-up) negativity thought intrusions.
